# Injectable, Anti-Cancer Drug-Eluted Chitosan Microspheres against Osteosarcoma

**DOI:** 10.3390/jfb13030091

**Published:** 2022-07-10

**Authors:** Jiebing Zhao, Hao Tian, Fusheng Shang, Tao Lv, Dagui Chen, Jianjun Feng

**Affiliations:** 1 Department of Orthopedics, Shanghai Pudong Hospital, Fudan University Pudong Medical Center, Shanghai 201399, China; 19211330003@fudan.edu.cn (J.Z.); 19211330005@fudan.edu.cn (H.T.); 19111330003@fudan.edu.cn (T.L.); 2 Institute of Translational Medicine, Shanghai University, Shanghai 200444, China; shangmuye116688@163.com (F.S.); dagui1106@shu.edu.cn (D.C.); 3 Fudan Zhangjiang Institute, Fudan University, Shanghai 201203, China

**Keywords:** osteosarcoma, electrospray, chitosan microspheres, proliferation, migration, apoptosis

## Abstract

The purpose of this study is to fabricate different anti-cancer drug-eluted chitosan microspheres for combination therapy of osteosarcoma. In this study, electrospray in combination with ground liquid nitrogen was utilized to manufacture the microspheres. The size of obtained chitosan microspheres was uniform, and the average diameter was 532 μm. The model drug release rate and biodegradation rate of chitosan microspheres could be controlled by the glutaraldehyde vapor crosslinking time. Then the 5-fluorouracil (5-FU), paclitaxel (PTX), and Cis-dichlorodiammine-platinum (CDDP) eluted chitosan microspheres were prepared, and two osteosarcoma cell lines, namely, HOS and MG-63, were selected as cell models for in vitro demonstration. We found the 5-FU microspheres, PTX microspheres, and CDDP microspheres could significantly inhibit the growth and migration of both HOS and MG-63 cells. The apoptosis of both cells treated with 5-FU microspheres, PTX microspheres, and CDDP microspheres was significantly increased compared to the counterparts of control and blank groups. The anti-cancer drug-eluted chitosan microspheres show great potential for the treatment of osteosarcoma.

## 1. Introduction

Combinatorial therapy, in which multiple drugs with different targets are used, has been widely used to treat cancers [1,2,3]. It is capable of improving the therapeutic effects of the traditional approach of monotherapy and reducing side effects caused by monotherapy (e.g., cytotoxicity, drug resistance). To date, many multi-drug delivery devices have been developed, such as the core-sheath structured fibers [4] and particles [5,6], multilayer structured nanofiber membranes [7,8], the introduction of particles into fibers or hydrogels [9]; however, these forms of multi-drug release devices are not convenient to use. They need to be implanted into the body through surgery. Compared to these biomaterials with a preformed shape, injectable biomaterials with minimally invasive surgery have attracted more and more attention, which allows for more exact implantation into deep anatomic regions [10,11]. Nano/micro particle is one form of injectable biomaterials, and the anti-cancer drug eluted particles are suitable for injecting into tumor sites for local chemotherapy [12,13].

Chitosan has been demonstrated as an ideal drug carrier and possesses many advantages such as biodegradability, no toxicity and biocompatibility [14]. According to the property of drug delivery application, chitosan can be prepared in various forms, such as beads, films, microspheres, hydrogels and so on [14]. Considering the higher drug loading ability of microspheres due to their high specific surface area and injectable ability, chitosan-based microspheres have gained increasing attention in the field of drug delivery [15,16,17]. For example, chitosan microspheres alone or embedded into hydrogel can achieve a sustained release of the drug or a controlled release of the drug at the target site [16,17].

Emulsion technology has been widely used to fabricate nano/micro particles [18,19]. For example, Jian Yang et al. used the double-emulsion technique to fabricate PLGA nanoparticles [20]; however, this method needs to undergo a couple of complicated steps, and the obtained particles need to be washed to remove organic solvents which may cause undesirable toxic effects; moreover, the dispersion of the obtained particles is not good, and they are easy to stick together because of stronger Van der Waal forces of droplets than the repellent forces in the suspension [21]. Electrospray is another common technique used to manufacture solid or hollow nano/micro particles via a simple needle or a coaxial needle [22,23,24]. In addition, the size of obtained microspheres could be customized according to applications by regulating experimental parameters, such as applied voltage, the concentration of the sprayed solution, flow rate, and so on [24]. For example, this technique could be used to generate chitosan microspheres of a different size via changing the molecular weight of chitosan and enhanced mucoadhesive properties for inhalable application [25]. In addition, in order to prevent the loading drug from being released in an undesired target, core-shell microspheres were developed via coaxial electrospray [26].

**Figure 1:**
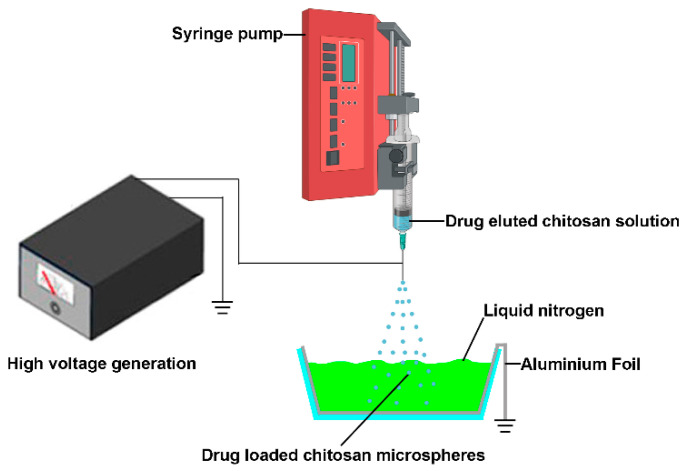
The schematic illustrates the manufacturing processes of drug-eluted chitosan microspheres by electrospray. The sprayed drug eluted chitosan microspheres were collected by grounded liquid nitrogen.

Although the electrospray technique exhibits promising prospects in the field of drug delivery, there are certain problems during the fabrication process. This method could directly deposit drug encapsulated particles to the substrate’s surface and adhere to a substrate’s surface (such as aluminum plates, stainless steel plates, steel plates covered with foil and the receiver solution) [24]; however, sometimes adhered microspheres tend to form aggregates, and it is not easy to detach these particles from collectors for further injection use due to their poor dispersion ability in the solution [27]. Jingwei Xie et al. used grounded liquid nitrogen to collect the particles made by electrospray. The liquid nitrogen was able to freeze and keep the original shape of obtained micro particles. The micro particles were obtained after the liquid nitrogen evaporates [28,29]; it inspires us that this technology could be used to fabricate injectable anti-cancer drug eluted microparticles for local chemotherapy (Figure 1). In this study, the 5-fluorouracil (5-FU), paclitaxel (PTX), and Cis-dichlorodiammine-platinum (CDDP) eluted chitosan microspheres were prepared, and the anti-cancer effects of these microparticles were examined in this study.

## 2. Materials and Methods

### 2.1. Materials

CS with a deacetylation level of 85% was purchased from Golden-Shell Pharmaceutical Co., Ltd. (Zhejiang, China) Bovine serum albumin (BSA), 5-fluorouracil (5-FU), paclitaxel (PTX), and Cis-dichlorodiammine-platinum (CDDP) were purchased from Sigma Aldrich (Shanghai, China). Acetic acid was purchased from Beijing Chemical Factory (Beijing, China). All chemicals used in this work were reagent grade. The electrospray apparatus included a needle and syringe, an aluminum foil immersed in liquid nitrogen, a grounding electrode, and a high-voltage direct-current power supply.

### 2.2. Fabrication of Chitosan Microspheres and Drug Eluted Chitosan Microspheres

To fabricate solid chitosan microspheres, 1.5 wt% of chitosan (Mw = 100,000) dissolved in a 2% acetic acid solution. Subsequently, the resulting solution was electrosprayed.

To fabricate drug eluted chitosan microspheres, 5-FU (50 mg), PTX (50 mg) and CDDP (50 mg) were respectively dissolved in DMF (50 mL) and then the 1.5 wt% of chitosan, along with 2% acetic acid, were added into the solution. 30 min sonication was applied to afford a homogenous solution for the following electrospray.

The optimal electrospray parameters were set as follows: v = 7 kV, flow rate = 2.0 mL/h, and distance between the needle tip to the aluminum foil collector of 20 cm [30]. The grounded liquid nitrogen was used to collect the chitosan microparticles. The collected chitosan microparticles were further crosslinked by the glutaraldehyde vapor for 10 and 20 min at room temperature, respectively [31,32]. The chitosan particles with or without crosslinking were put in a chemical hood for one week to remove the residual acetic acid and glutaraldehyde.

### 2.3. Drug Encapsulation Efficiency

The drug encapsulation of the microspheres was calculated according to a method described previously [33,34]. Briefly, 1 mg of dried drug-eluted chitosan microspheres was dissolved in 5 mL 1% HCl solution with an ultrasonic-assisted method. Thereafter the mixed solution was filtered and the filtrate was diluted with 5 mL of fresh 1% HCl solution to a fixed volume of 10 mL. The 5-FU and PTX drug content supernatant solution was then measured by UV spectroscopy at the wavelength of 265 nm or 228 nm. To determine the CDDP encapsulation efficiency, this solution was then evaporated and the dried residue was dissolved in a 1% HCl aqueous solution and the total platinum content was measured by ICP-MS. The drug encapsulation efficiency was then calculated using the following expressions.

$$\mathrm{Encapsulation}\;\mathrm{efficiency}\;(\%)\;=\;\frac{\mathrm{Weight}\;\mathrm{of}\;\mathrm{the}\;\mathrm{drug}\;\mathrm{in}\;\mathrm{microspheres}}{\mathrm{Weight}\;\mathrm{of}\;\mathrm{feeding}\;\mathrm{drug}}\;\times \;100\%$$

### 2.4. SEM Characterization

The chitosan micro particles were on the surface of conductive tape, then were sputter-coated with Pt for 5 min using an E102 ion sputtering apparatus (Hitachi, Tokyo, Japan) and observed by a scanning electron microscope at 25 kV (FEI, Quanta 200, Billerica, MA, USA).

### 2.5. Fabrication of Chitosan Microspheres Loaded with BSA and BSA Release Test

The BSA (bovine serum albumin) encapsulated chitosan microspheres were fabricated as follows. 1.5% chitosan and 0.5% BSA (Mw = 100,000) were dissolved in 2% acetic acid solution. The electrospray parameters used were the same as previously described. The grounded liquid nitrogen was used to collect the BSA-loaded microparticles, which were further crosslinked by the glutaraldehyde vapor for 10 and 20 min at room temperature, respectively [31,32]. Briefly, the BSA-loaded microparticles were placed at room temperature in a sealed desiccator where 6 mL of 25% aqueous glutaraldehyde solution were put to generate glutaraldehyde vapor for crosslink. The release of BSA was quantified by Pierce™ BCA Protein Assay Kit with a plate reader at 562 nm.

### 2.6. Degradation Test

The chitosan microparticles with or without crosslinking were weighted (weight o) and placed into lysozyme solution buffer at 37 °C with a final concentration of 800 mg/L. At each indicated time point, the residual chitosan particles were rinsed and freeze-dried, then the residual mass was measured (weight r). The residual mass (%) was calculated as the following equation. The residual mass (%) = (weight r/weight o) × 100%.

### 2.7. In Vitro Release Study

In vitro release studies were conducted to access the release profile of 5-FU, PTX and CDDP from the drug-eluted chitosan microspheres into media over a period of 30 days. 5 mg drug-loaded microspheres were placed in 2 mL PBS solution at pH = 7.4 and pH = 5.5 in a shaking incubator (170 rpm) at 37 °C. At preconditioned different time points, 1 mL liquid were removed for HPLC determination of 5-FU and PTX concentration and replaced with the same amount of fresh PBS solution. The amount of CDDP release was monitored by inductively couples’ plasma mass spectrometry.

### 2.8. CCK-8 Assay

The HOS cells and MG-63 cells were seeded into 96-well plates with a density of 2000 cells per well and incubated for 24 h. Then, 5-fluorouracil (5-FU), paclitaxel (PTX), and Cis-dichlorodiammine-platinum (CDDP) eluted chitosan microspheres with different concentrations (5, 15, 30, 45 and 60 μg) were added, respectively. Cells treated with empty chitosan microspheres were a blank group. The cells without any treatment were set as a control group. After 2 days of culture, 10 μL CCK-8 solution was added to each well, following the manufacturer’s instructions. Then, the absorbance was detected at 450 nm at different time points. All experiments were performed at least 3 times. The mean drug concentration required for 50% growth inhibition (IC50) was calculated.

### 2.9. Transwell Assay

Both HOS and MG-63 cells were placed in Transwell inserts (2 × 10^3^ cells/per well) and cultured with 100 μL serum-free DMEM in the 24-well cell culture well. Then 2 μg of 5-fluorouracil (5-FU), paclitaxel (PTX), and Cis-dichlorodiammine-platinum (CDDP) eluted chitosan microspheres were added to the upper chamber, respectively. Cells treated with empty chitosan microspheres were blank groups. The cells without any treatment were set as a control group. The lower chamber of the inserts was refreshed with DMEM containing 20% FBS. Following 24 h of incubation, cells that migrated to the bottom surface were fixed with methanol for 30 min. Subsequently, cells were stained with hematoxylin for 5 min. Finally, the invasive cells were observed and counted in five randomized regions under a light microscope.

### 2.10. Apoptosis Assay

Both HOS and MG-63 cells treated with different microspheres for 2 days were detached with trypsin and washed with PBS. Cells were subsequently centrifuged and stained with Annexin V-FITC/propidium iodide (Sigma-Aldrich; Shanghai, China) at room temperature. Finally, the apoptotic cells were evaluated by flow cytometry and repeated three times. Both the early and late stages of apoptosis were quantified.

### 2.11. Western Blot Assay

Both HOS and MG-63 cells treated with different microspheres for two days were lysed in the Lysis Buffer and then centrifugated (13,000× *g*, 30 min, 4 °C). 50 µg proteins were separated by 10% SDS-PAGE, then electrically transferred onto polyvinylidene difluoride (PVDF) membranes. Then, the PVDF membranes were blocked for one hour at room temperature containing 5% milk in Tris-buffered saline with 0.05% Tween 20. Membranes were separately incubated with Caspase-3 (Abcam, catalogue number: ab184787, 1:500), BCL-2 (Invitrogen, catalogue number: 33-6100, 1:500), BAX (Invitrogen, catalogue number: MA5-14003, 1:500), GAPDH (Abcam, catalogue number: ab9485, 1:1000). The secondary antibody conjugated with horseradish peroxidase was incubated at a 1:1000 dilution. Positive band intensities were visualized by utilizing the ECL detection system.

### 2.12. Statistical Analysis

All results were indicated as the mean ± S.D. One-way ANOVA followed by Turkey’s post hoc test were performed for statistical comparison using GraphPad Prism 8.0 software. Significance levels were set at * *p* < 0.05, ** *p* < 0.01 and *** *p* < 0.001.

## 3. Results and Discussion

### 3.1. The Fabrication and Physical Characterization of Drug Eluted Chitosan Microspheres

**Figure 2:**
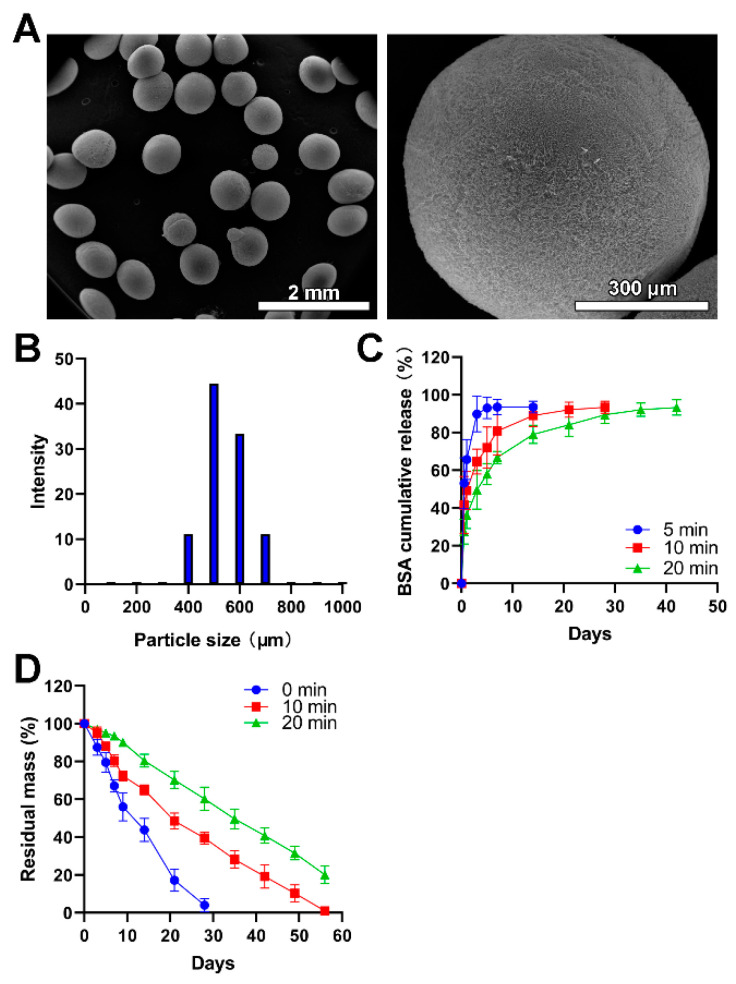
The fabrication and physical characterization of chitosan microspheres. (**A**) SEM images with low-resolution and high-resolution. (**B**) The size distribution of microspheres. (**C**) The BSA release profiles of chitosan microspheres (Blue), chitosan microspheres with glutaraldehyde vapor crosslinking for 10 min (red), and chitosan microspheres with glutaraldehyde vapor crosslinking for 20 min (green). (**D**) The in vitro lysozyme degradation behaviors of chitosan microspheres (Blue), chitosan microspheres with glutaraldehyde vapor crosslinking for 10 min (red), and chitosan microspheres with glutaraldehyde vapor crosslinking for 20 min (green).

In the presented study, electrospray was utilized to fabricate the chitosan microspheres. Compared with microspheres fabricated via emulsion technology, this method did not incorporate any organic solvent which could lead to undesired toxicity once adhered to microspheres during the fabrication process [35]. During the collection of these microspheres, the lower temperature generated by liquid nitrogen is beneficial to the shape maintenance of obtained microspheres and pharmacological activities of encapsulated drugs, especially for the delivery of nucleic acids and proteins. As shown in Figure 2A, the size of obtained chitosan microspheres was uniform. The average size was 532 μm, mainly in the range of 450–650 μm, which accounted for 77.77% (Figure 2B). The size of obtained microspheres could be adjusted by changing the concentration of the electrosprayed solution and the voltage applied to the droplets. In addition, a larger average size means more drugs or larger molecules encapsulated into the carrier, which could be hardly achieved by nanoparticles like liposomes [36]. 

The drug encapsulation efficiency of 5-FU, PTX and CDDP was 72.4 ± 4.3%, 64.6 ± 3.9% and 54.8 ± 5.9%, respectively. There were no significant differences in the drug trapping efficiency and the reproducibility of these procedures was considered good. Considering the larger size of particles, higher encapsulation efficiency could be understandable since the diffusion of the drug into the external phases was hindered due to increased length of the diffusion pathways [37].

The BSA was used as a model drug to characterize the drug release profile of chitosan microspheres since the release of BSA could be easily detected and precisely quantified. As shown in Figure 2C, the BSA showed a burst release in the BSA-loaded chitosan microspheres without glutaraldehyde vapor crosslinking, and it completed release within 3 days. The degradation of the chitosan matrix and the porosity of microspheres exert great influence on the rate of drug release [38]. This result was consistent with previous literature [39] where drug-loaded chitosan microspheres without chemical crosslinking can hardly achieve sustained release of drugs while crosslinking by glutaraldehyde could ameliorate this problem. The BSA showed a burst release in initiate and following a sustained release in the chitosan microspheres with glutaraldehyde vapor crosslinking. The sustained release property was further prolonged in the chitosan microspheres with glutaraldehyde vapor crosslinking for 20 min compared to it with glutaraldehyde vapor crosslinking for 10 min. In addition, microspheres with different porosity, which allows the diffusion of drugs to exhibit different release kinetics. The reduction of porosity means prolonged drug release time. Compared with other electrosprayed microspheres collected at room temperature [40,41], liquid nitrogen generated more regular and microporous particles, allowing the diffusion of drugs.

The in vitro lysozyme degradation revealed that the chitosan microspheres without crosslinking wholly degraded within four weeks; moreover, the chitosan microspheres with glutaraldehyde vapor crosslinking for 10 min completely degraded within 8 weeks. There was 22.13% residual mass of chitosan microspheres with glutaraldehyde vapor crosslinking for 20 min after 8 weeks of in vitro lysozyme degradation (Figure 2D); this result was in line with previous literature [31]. More crosslinking time did not influence microspheres’ degradability, but prolonged the degradation time, thus achieving controlled release of drugs encapsuled into the microspheres.

### 3.2. Anticancer Drugs Loading and Release Profiles

**Figure 3:**
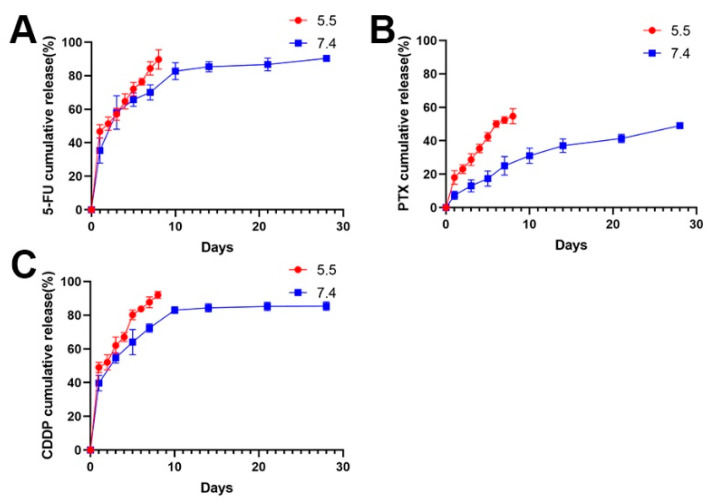
(**A**) The release profile of 5-FU loaded chitosan microspheres at pH 5.5 and pH 7.4. (**B**) The release profile of PTX loaded chitosan microspheres at pH 5.5 and pH 7.4. (**C**) The release profile of CDDP loaded chitosan microspheres at pH 5.5 and pH 7.4.

Following this, the 5-FU chitosan microspheres, PTX chitosan microspheres, and CDDP microspheres were prepared using the same method. The release of drug-eluted chitosan microspheres exhibited a two-stage profile: an initial burst release in the first several days and sustained release in the following stage. As shown in Figure 3, the 5-FU and CDDP showed a burst release at the beginning, and completed release around 10 days (Figure 3A,B); this is a common phenomenon of drug-loaded microspheres [42]. The initial rapid release can be attributed to the diffusion of drugs adhered to the surface of microspheres. After that, with the slow degradation of microspheres, drugs wrapped inside the microspheres exhibited sustained release profile; moreover, the PTX showed a slow release because of strong hydrophobicity of PTX (Figure 3C). 

Considering the acidic environment in osteosarcoma, the drug release performances of drug-eluted microspheres were investigated at pH 5.5 (Figure 3). Obviously, the release rate of drugs was faster at pH 5.5 than that at pH 7.4 in the PBS solution. It is understandable that drugs can be easily released form expanded walls of microspheres due to faster swelling process of chitosan under acidic environments [37,43].

### 3.3. Growth Inhibition Effects of Anticancer Drug-Loaded Chitosan Microspheres on Osteosarcoma Cells

**Figure 4:**
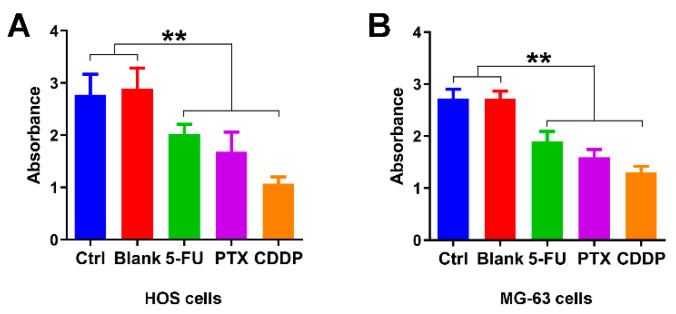
Growth inhibition effects of anticancer drug-loaded chitosan microspheres on osteosarcoma cells. (**A**) The absorbance of HOS cells treated with empty chitosan microspheres (Blank), 5-FU microspheres, PTX microspheres and CDDP microspheres for 3 days, the HOS cells without any treatment as control. (**B**) The absorbance of MG-63 cells treated with empty chitosan microspheres, 5-FU microspheres, PTX microspheres and CDDP microspheres for 3 days, the MG-63 cells without any treatment as control. ** *p* < 0.01.

We first examine the growth inhibition effects of 5-FU chitosan microspheres, PTX chitosan microspheres, and CDDP chitosan microspheres. The half-maximal inhibitory concentration (IC50) values of 5-FU CS microspheres, PTX CS microspheres, and CDDP CS microspheres were 25.3, 28.9 and 29.5 μg/mL, respectively. Two osteosarcoma cell lines were selected (e.g., HOS cells, MG-63 cells) for the in vitro study. As illustrated in Figure 4A, the absorbance of HOS cells treated with empty chitosan microspheres was closed to the control group. The absorbance of HOS cells with the treatment of 5-FU microspheres, PTX microspheres, and CDDP microspheres for 3 days was significantly decreased compared with the control and blank groups. Similarly, the absorbance of MG-63 cells with the treatment of 5-FU microspheres, PTX microspheres, and CDDP microspheres for 3 days was also lower compared to the control and blank groups (Figure 4B); these results suggested that chemotherapeutic drugs released from chitosan microspheres could effectively induce anti-proliferative effect on osteosarcoma cell lines.

### 3.4. Migration Inhibition Effects of Anticancer Drug-Loaded Chitosan Microspheres on Osteosarcoma Cells

**Figure 5:**
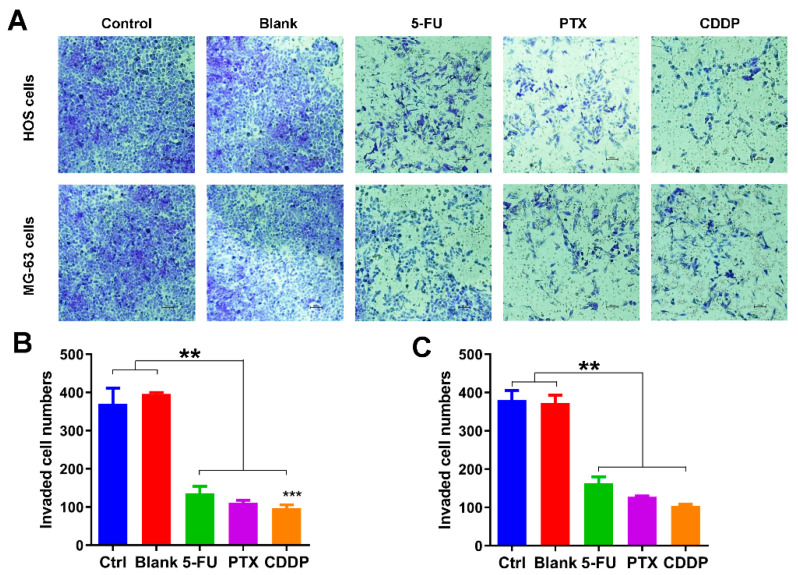
Migration inhibition effects of anticancer drug-loaded chitosan microspheres on osteosarcoma cells. (**A**) The invaded HOS and MG-63 cells exposed to empty chitosan microspheres (Blank), 5-FU microspheres, PTX microspheres and CDDP microspheres for 3 days, the HOS and MG-63 cells without any treatment as control. The quantification of migrated HOS cells (**B**) and MG-63 cells (**C**) of control, blank, 5-FU microspheres, PTX microspheres and CDDP microspheres groups. Significance levels were set at ** *p* < 0.01 and *** *p* < 0.001.

Following this, we also explored the effects of anticancer drug-loaded chitosan microspheres on the migration of osteosarcoma cells. As shown in Figure 5A, the transwell migration assay was used to evaluate the impact of anticancer drug-loaded chitosan microspheres on the migration of osteosarcoma cells. Less osteosarcoma cells (HOS and MG-63 cells) were found on the lower surface of the membrane in 5-FU microspheres, PTX microspheres, and CDDP microspheres treated groups, indicating that cells exposed to drug-eluted chitosan microspheres have difficulty in invading the membrane. The quantification of migrated HOS and MG-63 cells exposed to 5-FU microspheres, PTX microspheres, and CDDP microspheres was much less than the control and blank groups (Figure 5B,C). These results indicated that the drug-eluted chitosan microspheres displayed the great potential in preventing the migration of osteosarcoma cells.

### 3.5. Apoptosis Promotion Effects of Anticancer Drug-Loaded Chitosan Microspheres on Osteosarcoma Cells

**Figure 6:**
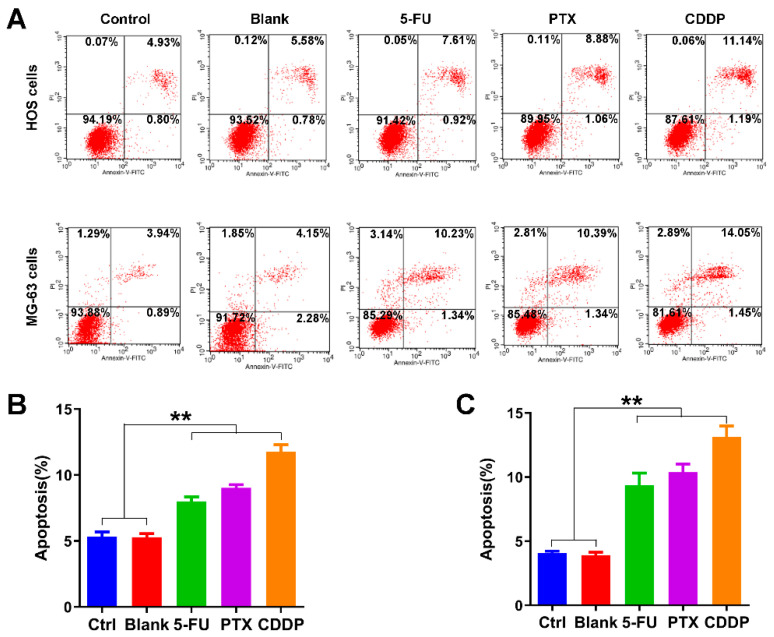
Apoptosis promotion effects of anticancer drug-loaded chitosan microspheres on osteosarcoma cells. (**A**) The flow cytometry apoptosis analysis of HOS cells and MG-63 cells exposed to empty chitosan microspheres (Blank), 5-FU microspheres, PTX microspheres and CDDP microspheres for 3 days, the HOS and MG-63 cells without any treatment as control. The apoptotic rate (%) quantification of HOS cells (**B**) and MG-63 cells (**C**) of control, blank, 5-FU microspheres, PTX microspheres and CDDP microspheres groups. Significance levels were set at ** *p* < 0.01.

We further examined the effects of anticancer drug-loaded chitosan microspheres on the apoptosis of osteosarcoma cells. As shown in Figure 6A, the average apoptotic rate of HOS cells of control, blank, 5-FU microspheres, PTX microspheres, and CDDP microspheres groups was 5.33%, 5.28%, 8.01%, 9.03%, and 11.75%, respectively. The average apoptotic rate of MG-63 cells of control, blank, 5-FU microspheres, PTX microspheres, and CDDP microspheres groups was 4.07%, 3.97%, 9.36%, 10.38%, and 13.14%, respectively. The apoptotic rate of HOS cells and MG-63 cells exposed to 5-FU microspheres, PTX microspheres, and CDDP microspheres were significantly higher than the control and blank groups (Figure 6B,C). 

**Figure 7:**
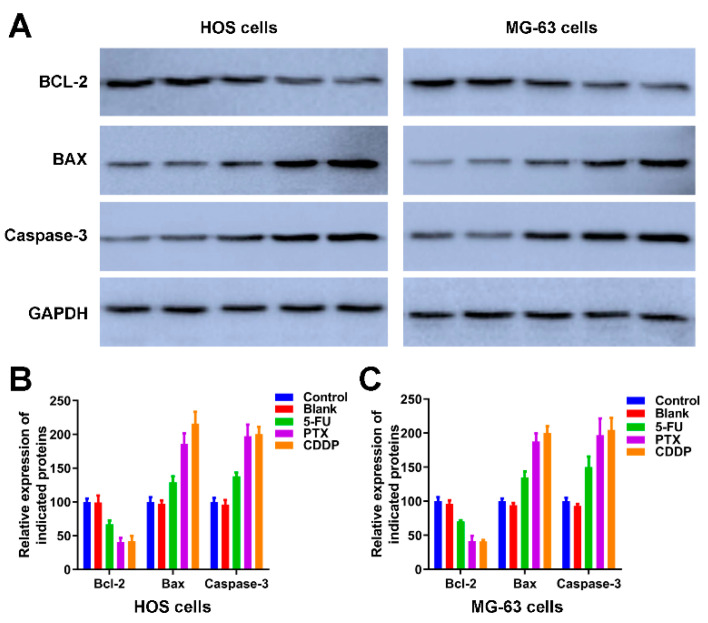
The effects of anticancer drug-loaded chitosan microspheres on the expression of apoptotic proteins on osteosarcoma cells. (**A**) The expression of apoptotic proteins including BCL-2, BAX and Caspase-3 on HOS and MG-63 cells of control, blank, 5-FU microspheres, PTX microspheres and CDDP microspheres groups. The quantification of the expression of apoptotic proteins including BCL-2, BAX and Caspase-3 on HOS cells (**B**) and MG-63 cells (**C**) of control, blank, 5-FU microspheres, PTX microspheres and CDDP microspheres groups.

We further detected the expression of apoptotic-related proteins, including Bcl-2, BAX and Caspase-3 [44] in Figure 7. As an anti-apoptotic protein, the expression trend of Bcl-2 is negatively correlated with apoptosis. The expression of Bcl-2 in HOS cells and MG-63 cells of control and blank groups was significantly higher than the Bcl-2 in HOS and MG-63 cells exposed to 5-FU microspheres, PTX microspheres, and CDDP microspheres. The BAX and Caspase-3 are apoptotic proteins, the band intensities of BAX and Caspase-3 in both HOS and MG-63 cells exposure to 5-FU microspheres, PTX microspheres, and CDDP microspheres were dramatically increased compared to the HOS cells and MG-63 cells of control and blank groups (Figure 7A). The quantification of Figure 7A further revealed the 5-FU microspheres, PTX microspheres, and CDDP microspheres could accelerate osteosarcoma cells’ apoptosis.

Taken together, all these results indicated that drug-eluted chitosan microspheres possessed great potential in cancer therapy; however, there are several limitations within the present study. First, considering the acidic tumor microenvironment, we should conduct the in vitro release study in an acidic environment. Second, more in vivo models such as a subcutaneous osteosarcoma xenograft model or an osteosarcoma xenograft model should be performed to verify the therapeutic efficiency of drug-eluted chitosan microspheres.

## 4. Conclusions

In this study, the electrospray in combination with ground liquid nitrogen was utilized to manufacture the microspheres. The 5-FU, PTX, and CDDP eluted chitosan microspheres were prepared. We found the 5-FU microspheres, PTX microspheres, and CDDP microspheres could significantly inhibit HOS cells’ growth and migration and MG-63 cells; moreover, the apoptosis of HOS cells and MG-63 cells treated with 5-FU microspheres, PTX microspheres, and CDDP microspheres was significantly increased compared to the HOS cells and MG-63 cells of control and blank groups. The anti-cancer drugs eluted chitosan microspheres show great potential for the treatment of osteosarcoma.

## Data Availability

Not applicable.
